# Microglia sustain anterior cingulate cortex neuronal hyperactivity in nicotine-induced pain

**DOI:** 10.1186/s12974-023-02767-0

**Published:** 2023-03-21

**Authors:** Dan-dan Long, Yu-zhuo Zhang, An Liu, Liang Shen, Hong-rui Wei, Qian-qian Lou, Shan-shan Hu, Dan-yang Chen, Xiao-qing Chai, Di Wang

**Affiliations:** 1 Pain Clinic, Department of Anesthesiology, The First Affiliated Hospital of USTC, Division of Life Sciences and Medicine, University of Science and Technology of China (USTC), Hefei, 230001 China; 2 Institute of Clinical Pharmacology, Anhui Medical University, Hefei, 230032 China; 3 Department of Physiology, School of Basic Medical Sciences, Anhui Medical University, Hefei, 230032 China; 4 Department of Neurobiology, Division of Life Sciences and Medicine, University of Science and Technology of China, Hefei, 230001 China; 5 Department of Clinical Laboratory, The First Affiliated Hospital of USTC, Division of Life Sciences and Medicine, University of Science and Technology of China (USTC), Hefei, 230001 China

**Keywords:** Chronic nicotine, Pain, Anterior cingulate cortex, Microglia, Glutamatergic neurons, CX3CL1

## Abstract

**Background:**

Long-term smoking is a risk factor for chronic pain, and chronic nicotine exposure induces pain-like effects in rodents. The anterior cingulate cortex (ACC) has been demonstrated to be associated with pain and substance abuse. This study aims to investigate whether ACC microglia are altered in response to chronic nicotine exposure and their interaction with ACC neurons and subsequent nicotine-induced allodynia in mice.

**Methods:**

We utilized a mouse model that was fed nicotine water for 28 days. Brain slices of the ACC were collected for morphological analysis to evaluate the impacts of chronic nicotine on microglia. In vivo calcium imaging and whole-cell patch clamp were used to record the excitability of ACC glutamatergic neurons.

**Results:**

Compared to the vehicle control, the branch endpoints and the length of ACC microglial processes decreased in nicotine-treated mice, coinciding with the hyperactivity of glutamatergic neurons in the ACC. Inhibition of ACC glutamatergic neurons alleviated nicotine-induced allodynia and reduced microglial activation. On the other hand, reactive microglia sustain ACC neuronal excitability in response to chronic nicotine, and pharmacological inhibition of microglia by minocycline or liposome-clodronate reduces nicotine-induced allodynia. The neuron-microglia interaction in chronic nicotine-induced allodynia is mediated by increased expression of neuronal CX3CL1, which activates microglia by acting on CX3CR1 receptors on microglial cells.

**Conclusion:**

Together, these findings underlie a critical role of ACC microglia in the maintenance of ACC neuronal hyperactivity and resulting nociceptive hypersensitivity in chronic nicotine-treated mice.

**Supplementary Information:**

The online version contains supplementary material available at 10.1186/s12974-023-02767-0.

## Background

Tobacco smoking serves as an important vehicle for nicotine delivery in humans, and nicotine is the primary reinforcing component driving tobacco addiction. Chronic nicotine exerts a reinforcing effect through repeated activation of nicotinic acetylcholine receptors (nAChRs), which are mainly expressed in dopamine neurons in the ventral tegmental region of the midbrain [1, 2]. Concurrently, chronic exposure to nicotine produces profound changes in human physiology. In addition to studies showing that the risks of respiratory and cardiovascular diseases are higher in smokers than in nonsmokers, the epidemiological and clinical evidence strongly suggests an association between cigarette smoking and the incidence and severity of chronic painful conditions [3, 4]. In the laboratory, several animal studies have demonstrated that chronic nicotine elicits stable, persistent mechanical allodynia in rodents that further exacerbates coexisting painful conditions [5]. However, directly linking the cellular effects of chronic nicotine to modifications of the pain perception-related neural system has been thought to be multifaceted and elusive.

Microglia are highly specialized resident macrophage-like cells that act as homeostatic sensors to monitor and sustain the balance of the microenvironment within the central nervous system (CNS) [6]. Their constant surveillance of the brain microenvironment enables microglia, characterized by extremely low thresholds, to be highly active in their continued response to any type of brain homeostasis disorder [7, 8]. Once ramified microglia transform into reactive states, reactive microglia exhibit substantial changes by altering their own morphology and number in diverse ways depending on the type of stimuli they sense. Microglia in the nucleus accumbens (NAc), which is a midbrain limbic region involved in the rewarding effects of nicotine, have been demonstrated to experience a substantial morphological change during nicotine dependence and withdrawal [9–11]. On the other hand, despite some disagreements in the literature [12], the majority of available studies provide compelling evidence that microglia are involved in pain modulation [13–15]. However, the central mechanisms that support a role for microglia in the development of chronic nicotine-induced pain have not been investigated.

The anterior cingulate cortex (ACC) is a critical area for the integration of nociceptive perception and emotional responses in chronic pain [16, 17]. In human studies, magnetic resonance imaging (MRI) has shown that the ACC is the most consistently activated region in patients with chronic pain [18]. Consistent with brain imaging data, the inhibition of hyperexcitable pyramidal neurons in the ACC has been reported to produce analgesia in experimental rodent models of inflammatory and neuropathic pain [19, 20]. The central mechanism underlying chronic pain is usually linked to microglial activation at both spinal and supraspinal sites (including the ACC). Our [19] and other works [21–23] indicated that reactive microglia in the ACC, in line with neuronal hyperexcitability, contributed to central neuroadaptation in chronic painful conditions, although the mechanistic details of the reciprocal interactions between neurons and microglia are not well elucidated. In particular, the ACC is also involved in reward processing and addiction. Compulsive use of the addictive substances is related to increased neural activity and structural abnormalities in the ACC [24, 25]. Therefore, this study aims to determine whether and how chronic nicotine elicits nociceptive hypersensitivity in mice through altering ACC microglial morphology and function as well as neuronal activity.

## Materials and methods

### Animals

All animal protocols were approved by the Animal Use and Ethics Committee, University of Science and Technology of China. Male C57BL/6J mice were purchased from Jackson Laboratory. In these experiments, all mice were used at 8–10 weeks of age, weighing approximately 25 g. All mice were kept on a 12-h light–dark cycle (lights on at 7 am) at a comfortable temperature (23–25 ℃) with free access to water and food. The animals were acclimatized to the environment for a week before the experiment.

### Animal models

The mice were randomly divided into 2 groups: the vehicle group and the nicotine group. Mice in the nicotine group were administered nicotine with L-tartaric acid (HuaXia, Chengdu, China) in their drinking water. Mice in the vehicle group drank tap water (pH 7.0) with l-tartaric acid. The following treatment schedule was used for nicotine and tartaric acid (in μg/mL), respectively: Day 1–2 (50,75), Day 3–4 (100,150), Day 5 and beyond (200, 300) [26]. The subsequent doses were maintained at 300 μg/mL, and the water was changed every 2–3 days. Saccharin sodium (3 mg/mL, Macklin, China) was dissolved in tap water to mask the bitter taste of nicotine. Mice were treated for 28 days prior to experimentation.

### Pain-like behaviour test

#### Von Frey filament test

The paw withdrawal threshold (PWT) of the left hind plantar surface of mice was measured using von Frey filaments (von Frey filaments, Stoelting Inc., USA). Before the test, the mice were placed alone in clear plastic boxes and placed on a wire mesh elevated platform to acclimate for at least 2 days, 30 min a day. Before modelling, all mice were tested for the basic threshold of nociceptive behaviour, and mice with abnormal basic nociceptive thresholds were excluded. On the testing day, after acclimatization for 30 min, mechanical allodynia was tested by von Frey filaments in ascending order (0.02, 0.04, 0.07. 0.16, 0.4, 0.6, and 1.0 g) to stimulate the plantar surface of the left hind paw [27]. The minimal force filament that induced the mice to present a brisk paw withdrawal, flinching, or licking was taken as the mechanical response threshold. If there was no positive nociceptive response, a filament with a greater force was applied, and the measurement was repeated three times to obtain an average threshold. Each measurement was spaced at least 5 min apart to prevent aversion from frequent stimuli.

#### Hargreaves test

Mice were placed on a glass plate at a constant temperature of 30 ℃ and separated with a transparent plastic chamber. The mice were acclimatized for at least two days for 30 min each before testing. The Hargreaves test was used to assess the thermal nociceptive threshold. On the testing day, after acclimatization for 30 min, the thermal nociceptive threshold of the left hind paw was measured by focusing a beam of light on the plantar surface using the Hargreaves apparatus (IITC Life Science, USA). The basal paw withdrawal latency (PWL) was adjusted to 8–15 s and the thermal laser stimulation on the paw lasted for only 20 s to avoid potential tissue damage. The heat stimulation was repeated three times at an interval of at least 5 min for each paw and the mean was calculated.

### Immunohistochemistry and imaging

Mice were deeply anaesthetized by intraperitoneal injection of sodium pentobarbital (50 mg/kg) and then perfused with 0.9% saline followed by 4% (w/v) paraformaldehyde. After perfusion, the brain was carefully removed and postfixed in 4% PFA at 4 °C for at least 24 h and then immersed in 20% and 30% sucrose solution at 4 ℃ for 2‒3 days for dehydration until isotonic. Coronal sections (40 μm) were prepared using a cryostat microtome system at – 20 ℃, and the sections were immersed in antifreeze solution and stored at − 20 ℃. For immunohistochemistry, the ACC brain slices were first incubated in 0.3% (v/v) Triton X-100 for 30 min and then incubated with 10% donkey serum for 1 h at room temperature to block nonspecific reactions, followed by incubation with primary antibodies diluted in blocking solution (0.3% Triton X-100, 10% donkey serum in PBS) at 4 °C for 24 h. The primary antibodies included anti-ionized calcium-binding adapter molecule 1 (Iba-1) (1:500, rabbit, Wako and 1:500, goat, Abcam), anti-Arginase (1:200, mouse, Abcam), anti-iNOS (1:100, rabbit, Abcam), anti-c-Fos (1:500, rabbit, Santa Cruz), anti-glutamate (1:500, rabbit, Sigma; 1:250, mouse, Sigma), anti-GABA (1:500, mouse, Sigma). After washing with PBS (3 × 5 min), the corresponding fluorophore-conjugated secondary antibodies (1:500, Invitrogen) were incubated with brain slices for 2 h at room temperature. At the last stage, the slices were incubated with 4,6-diamidino-2-phenylindole (DAPI; 1:2000, Sigma) for 5 min washed with PBS three times and mounted for imaging. The fluorescence signals were visualized using a Leica DM2500 camera and a Zeiss LSM980 microscope. All the experimental details are described elsewhere [19].

### In vivo fibre-optic calcium recording

Calcium signals were recorded by using fibre photometry. As indicated also by previous paper [28], mice were treated with 5% (w/v, i.p.) chloral hydrate and fixed in a stereotactic frame (RWD, Shenzhen, China). rAAV-CaMKIIα- GCaMP6m-EGFP-WPRE-pA (AAV 2/9, 5.04 × 10^12^ vg/mL, BrainVTA) was injected into the ACC (anterior posterior [AP] from bregma: 0.98 mm, medial lateral [ML] from the midline: 0.34 mm, dorsal ventral [DV] from the brain surface: − 1.50 mm) at a volume of 200 nL. An optical fibre (Inper, Hangzhou, China) was implanted in the same place and fixed with dental cement and glue. After 3 weeks, the virus was successfully expressed and the signal was recorded. To record fluorescence signals from GCaMP6m, light from a 470-nm LED was bandpass filtered (470/10 nm), collimated, reflected by dichroic mirrors, focused using a 20 × objective, and then delivered at a power of 25–40 μW at the tip of the fibre optic cannula. The fluorescence emitted by GCaMP6m was filtered by bandpass (525/40 nm) and focused on the sensor of a CMOS camera. The end of the fibre was imaged at a frame rate of 60 fps with InperSignal, and the mean value of the ROI at the end-face of the fibre was calculated using InperPlot software. To serve as an isosbestic control channel, LED light at 410 nm was bandpass filtered (410/10 nm) and transmitted alternately with LED light at 470 nm. GCaMP6m fluorescence intensity before and during punctate mechanical stimulation (von Frey filaments) was then recorded. We used a video camera to record behaviour. The fluorescence change value (ΔF/F) was obtained by calculating (F − F0)/F0, and the signal before the stimulus presentation 5 s was defined as the baseline. Data were analysed by InperPlot software (Inper Technology, Hangzhou).

### In vitro electrophysiological recordings

#### Brain slice preparation

For acute brain slice preparation, mice were deeply anaesthetized with pentobarbital sodium (2% w/v, i.p.) and subsequently intracardially perfused with ice-cold oxygenated *N*-methyl-d-glucamine artificial cerebrospinal fluid (NMDG ACSF) that contained 93 mM *N*-methyl-d-glucamine (NMDG), 1.2 mM NaH_2_PO_4_, 2.5 mM KCl, 20 mM *n*-2-hydroxyethylpiperazine-*N*-2-ethanesulfonic acid (HEPES), 30 mM NaHCO_3_, 2 mM thiourea, 25 mM glucose, 3 mM Na-pyruvate, 5 mM Na-ascorbate, 10 mM MgSO_4_, 0.5 mM CaCl_2_, and 3 mM glutathione (GSH). Then, the brain was quickly removed from the skull. Coronal sections containing ACC (300 μm) were sectioned with a vibratome microtome (VT1200s, Leica) at 0.18 mm/s, incubated in NMDG ACSF at 33 ℃ for 10–12 min, and then transferred to HEPES artificial cerebrospinal fluid (ACSF) that contained 2.5 mM KCl, 92 mM NaCl, 30 mM NaHCO_3_, 20 mM HEPES, 1.2 mM NaH_2_PO_4_, 2 mM thiourea, 25 mM glucose, 3 mM Na-pyruvate, 5 mM Na-ascorbate, 2 mM MgSO_4_, 2 mM CaCl_2_, and 3 mM GSH at 25 °C for at least 1 h. The brain slices were transferred to a slice chamber (Warner Instruments) for whole-cell recording and were continuously perfused at a rate of 3 ml/min with oxygenated standard ACSF solution (32 °C) that contained 2.4 mM CaCl_2_, 3 mM KCl, 129 mM NaCl, 20 mM NaHCO_3_, 1.3 mM MgSO_4_, 1.2 mM KH_2_PO_4_, and 10 mM glucose. An in-line solution heater was used to maintain the temperature of the standard ACSF (TC-344B, Warner Instruments, USA).

### Whole-cell patch-clamp recordings

Neurons in the ACC were visualized with a 40 × water immersion objective on an upright microscope (BX51WI, Olympus, Japan) equipped with interference contrast (IR/DIC) and an infrared camera connected to the video monitor. CaMKIIα-Cre::Ai14 mice in which tdTomato specifically labels glutamatergic neurons were used. We used a patch-clamp amplifier (MultiClamp 700B Amplifier, Digidata 1440Aanalog-to-digital converter, USA) and pClamp 10.7 software (Axon Instruments/Molecular Devices, USA) for whole-cell patch-clamp recording. Patch pipettes were pulled from borosilicate glass capillaries (VitalSense Scientific Instruments Co., Ltd., Wuhan, China) with an outer diameter of 1.5 mm on a four-stage horizontal puller (P1000, Sutter Instruments, USA). Voltage-clamp recording was performed with glass pipette filling containing 10 mM HEPES, 130 mM k-gluconate, 5 mM KCl, 2 mM MgCl_2_, 0.6 mM EGTA, and 0.3 mM Na-GTP (osmolarity: 285–290 mOsm/kg, pH: 7.2). The threshold current for firing was defined as the minimum strength of current injection required to elicit at least one or two spikes. All recordings were Bessel-filtered at 2.8 kHz and sampled at 100 kHz. Throughout the recording, only neurons with series resistance < 30 MΩ were used for analysis.

### Chemogenetic manipulation

Mice were anaesthetized with 5% (w/v, i.p.) chloral hydrate and fixed in a stereotactic frame (RWD, Shenzhen, China). A 200 nL volume of virus (rAAV-CaMKIIα-hM4Di-mCherry [AAV 2/9, 5.85 × 10^12^ vg/mL, BrainVTA] or rAAV-CaMKIIα-mCherry [AAV 2/9, 2.69 × 10^12^ vg/mL, BrainVTA]) was injected into the ACC (AP: 0.98 mm; ML: 0.34 mm; DV: − 1.50 mm) using calibrated glass microelectrodes connected to an infusion pump (RWD, Shenzhen, China) at a rate of 30 nL/min. Behavioural tests were performed at least 3 weeks after viral injection. For chemogenetic manipulation, the chemical ligand clozapine-N-oxide (CNO) (5 mg/kg, MCE, Monmouth Junction, NJ, USA) was intraperitoneally injected into these mice under isoflurane anaesthesia. Behaviour tests were then carried out at least 30 min later [19].

### Morphological analysis

In agreement with other published work [29], for morphometric analysis, confocal images of Iba1-positive cells were visualized and acquired by a confocal laser-scanning microscope (Carl Zeiss LSM880, Germany) with a 40 × objective at 30-μm intervals along the z-axis. The confocal Z-stack image file was analysed using the function ''Calculate Diameter of Filaments from Image''. The diameter of counted events was set between 0.25 μm and 15 μm.

We modulated the “Starting Point” and “Seed Point Thresholds” of dendrites according to the actual size, then we selected “Remove Seed Points Around Starting Points” and set the diameter of “Sphere Regions” as 30 μm. The number of events was the number of myeloid cells in the image counted per mm3 according to the volume of the image. Subsequently, the threshold of the dendrites was adjusted using the spine “Points Diameter” step, and then the “Detect Spines” option was selected. Furthermore, after using the ''Dendrite length'' function from the statistics tab, the data was saved and used as a measure of microglial morphology, such as the number of endpoints and process length. Three images were randomly picked from each mouse, and the mean result was used for morphological analysis. Three-dimensional reconstruction of microglia was performed using IMARIS software.

### Fluorescence-activated cell sorting

As previously published work [30], mice were anaesthetized with an intraperitoneal injection of pentobarbital (2% w/v, i.p). Subsequently, mice were perfused intracardially with 20 mL of 0.1 M cold Hanks’ balanced salt solution (HBSS), followed by a rapid collection of the ACC (6 pooled animals per N), which was washed with cold HBSS and chopped into small pieces on ice. Small tissue was mechanically homogenized using a 23G needle to produce single-cell suspension, which was filtered through a 70 μm cell strainer. After 70–30% Percoll gradient (Sigma, USA) separation, the single-cell suspension was isolated from the interface and filtered with a 200 μm nylon mesh prior to antibody staining. Microglia were labelled with CD11b-PC5.5 (1:20, Biolegend) and sorted by BD FACSAria III (BD, USA) for subsequent qPCR and immunohistochemistry experiments.

### Real-time PCR analysis

TRIzol reagent (Vazyme, Nanjing, China) was used to extract total RNA from isolated microglia of the ACC, which was quantified using NanoVue plus (GE, USA). Purity was assessed by the quotient 260/280 nm. Approximately 500 ng of total RNA was reverse transcribed using StarScript II First-strand cDNA Synthesis Mix with gDNA Remover (GenStar, Beijing, China) in 20 μL reactions following the manufacturer’s protocol. Quantitative real-time PCR (qPCR) for *TNF-α, IL-1β, IL-6, CX3CL1, Arg-1* and *iNOS* was performed on an Applied Biosystems StepOneTM Real-Time PCR System using 2 × TSINGKE Master qPCR Mix (SYBR Green I) (Tsingke Biotechnology, Beijing, China) [19].

Gene-specific primers were purchased from Tsingke Biotechnology. The primer sequences were as follows:

*TNF-α* (forward 5′-CCTGTAGCCCACGTCGTAG-3′,reverse 5′-GGGAGTAGACAAGGTACAACCC-3′);

*IL-1β* (forward 5′-TTCAGGCAGGCAGTATCACTC-3′,reverse 5′-GAAGGTCCACGGGAAAGACAC-3′);

*IL-6* (forward 5′-GCCAACATTTTATTTCCGGGA-3′,reverse 5′-CCACTGAGCATATTTCTCGGG-3′);

*CX3CL1* (forward 5′-ACGAAATGCGAAATCATGTGC-3′,reverse 5′-CTGTGTCGTCTCCAGGACAA-3′);

*Arg-1* (forward 5′-AAGAAAAGGCCGATTCACCT-3′,reverse 5′-CACCTCCTCTGCTGTCTTCC-3′);or *iNOS* (forward 5′-GACATTACGACCCCTCCCAC-3′,reverse 5′-ACTCTGAGGGCTGACACAAG-3′).

### Drug administration into the ACC

Mice were deeply anaesthetized with pentobarbital sodium (2% w/v, i.p.). We used a dental drill for craniotomy and then implanted a guide cannula (O.D.0.41 mm-27G/M3.5, RWD, Shenzhen, China) above the unilateral ACC (AP: 0.98 mm; ML: 0.34 mm; DV: − 1.30 mm). Modelling was started 21 days before catheterization. An injection cannula (O. D.0.20 mm-30G/M3.5, RWD, Shenzhen, China) with a PE tube was inserted into the guide cannula and drug or standard ACSF was injected for 1 min using a microinjector pump (RWD, Shenzhen, China) on Day 21 postmodelling. As previously described, a dosage of 10 mg/mL minocycline (500 nL, Aladdin, Shanghai, China) [19], a dosage of 5 mg/mL liposomes-clodronate (500 nL, Target Technology, Beijing, China) [31], a dosage of 0.5 ng/nL JMS-17-2 (100 nL, MedChemExpress, US) [32] were employed in the present study.

### Statistical analysis

The parametric data are expressed as the mean ± SEM, and nonparametric data are presented as the median (IQR). Histograms or QQ plots was used to assess whether the data conformed to a normal distribution. If the distribution was normal, GraphPad Prism version 8.0 (GraphPad Software, Inc., USA) was used for statistical analysis and graphing. The unpaired two tailed Student’s t test was used for comparisons between two groups. One-way analysis of variance (ANOVA) or two-way ANOVA followed by Bonferroni test was used for multiple comparisons. Otherwise, the nonnormally distributed data were analysed by nonparametric test. Significance levels are displayed as *p < 0.05, **p < 0.01, ***p < 0.001 and not significant (ns).

## Results

### Chronic nicotine exposure alters ACC microglial morphology

**Fig. 1:**
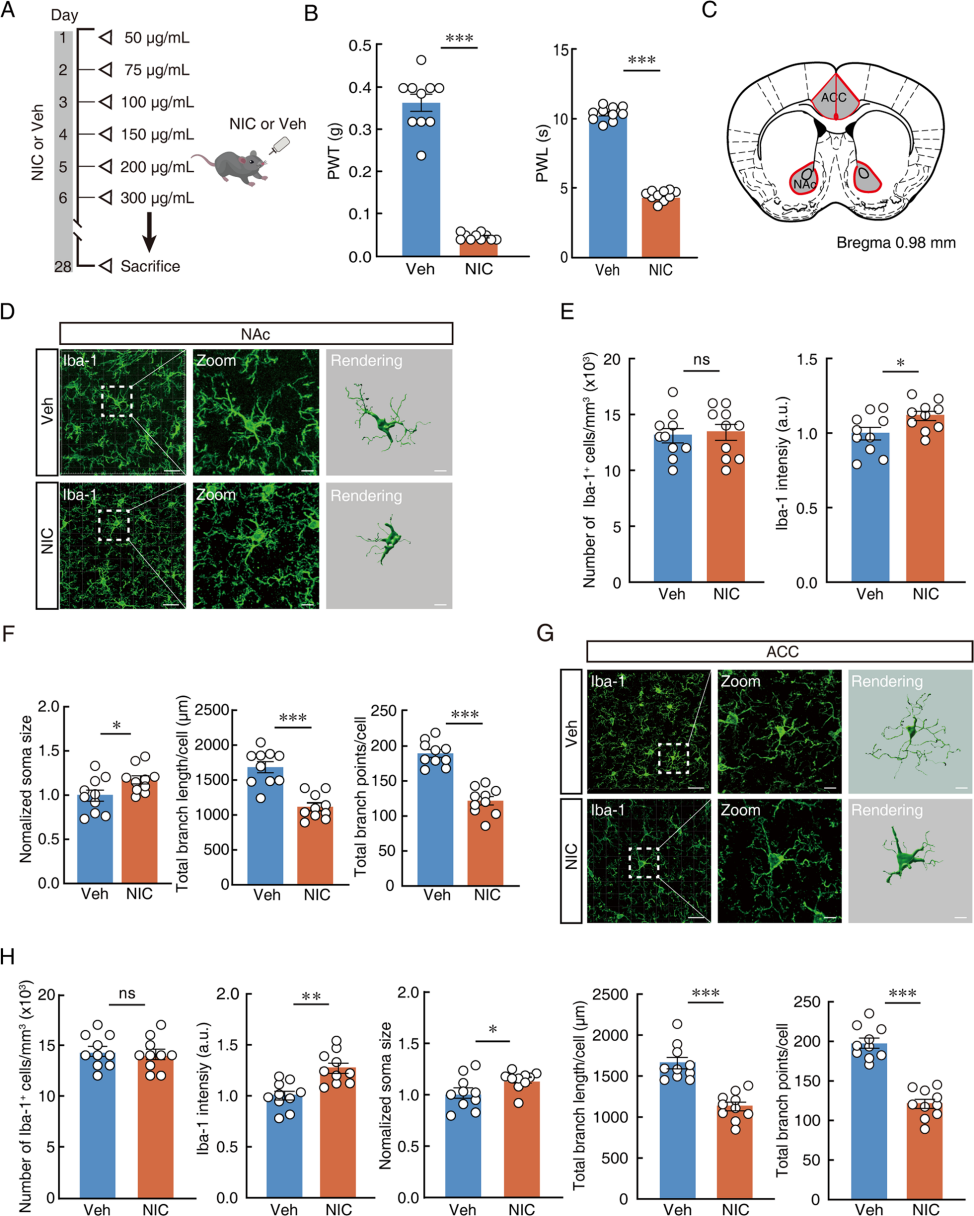
Chronic nicotine exposure alters ACC microglial morphology.** A** Schematic timeline of administration with L-tartaric acid and nicotine. **B** Changes of PWT and PWL in vehicle- and nicotine-treated mice **C** Representations from Paxinos & Franklin mouse atlas of regions of interest analysed. Anterior cingulate cortex (ACC) and Nucleus accumbens (NAc). **D** Representative imaging and 3D remodeling of Iba-1-labelled microglia in the Nucleus accumbens (NAc) of vehicle- and nicotine-treated mice after 28 days. Scale bars, 40 μm (overview) and 10 μm (Zoom and Rendering). **E** Quantification of Iba-1^+^ number and intensity in the NAc **F** Quantification of Iba-1^+^ soma size and Imaris-based semi-automatic quantification of Iba-1^+^ microglia morphometry in the NAc. **G** Representative imaging and 3D remodeling of Iba-1-labelled microglia in the anterior cingulate cortex (ACC) of vehicle- and nicotine-treated mice after 28 days. Scale bars, 40 μm (overview) and 10 μm (Zoom and Rendering). **H** Quantification of Iba-1^+^ number, intensity, soma size and Imaris-based semi-automatic quantification of Iba-1^+^ microglia morphometry in the ACC. Data were presented as mean ± SEM or Median (IQR). *p < 0.05; **p < 0.01; ***p < 0.001; *ns* not significant. Details of the statistical analyses are presented in Additional file 1: Table S1

Microglia exhibit substantial morphological and/or numerical changes once activated [33]. To evaluate both features during chronic nicotine exposure, we treated male mice with nicotine via their drinking water to induce nicotine dependence (Fig. 1A), as previously described [26, 34, 35]. Consistent with previously published findings [5, 36, 37], our results have shown that chronic exposure to nicotine produces stable, persistent allodynia in male mice. Compared to vehicle control group, mechanical and thermal thresholds were reduced in nicotine-treated mice (Fig. 1B). We performed morphological reconstruction of microglia by using Iba-1 staining and further assessed the changes in number and morphological processes. We used NAc microglia here as a control, since the results reported in previous studies have shown that microglia in the NAc, a midbrain limbic region related to the rewarding effects of nicotine, are activated in response to chronic nicotine in mice. In this experiment, NAc microglia underwent a series of morphological changes after mice were fed nicotine water (Fig. 1C–F), confirming previously published results. In addition to NAc reactive microglia, we found that microglia in the ACC from mice received nicotine and vehicle showed substantial differences in microglial morphology rather than cell number. Compared with the vehicle control, microglia in the ACC from nicotine-treated mice had different morphologies, with features of a larger cell area, shorter processes and decreased branch points (Fig. 1G, H). These results have shown that allodynia developed with a reactive status of ACC microglia in mice subjected to chronic nicotine.

### Reactive microglia in the ACC are required for nicotine-induced allodynia

**Fig. 2:**
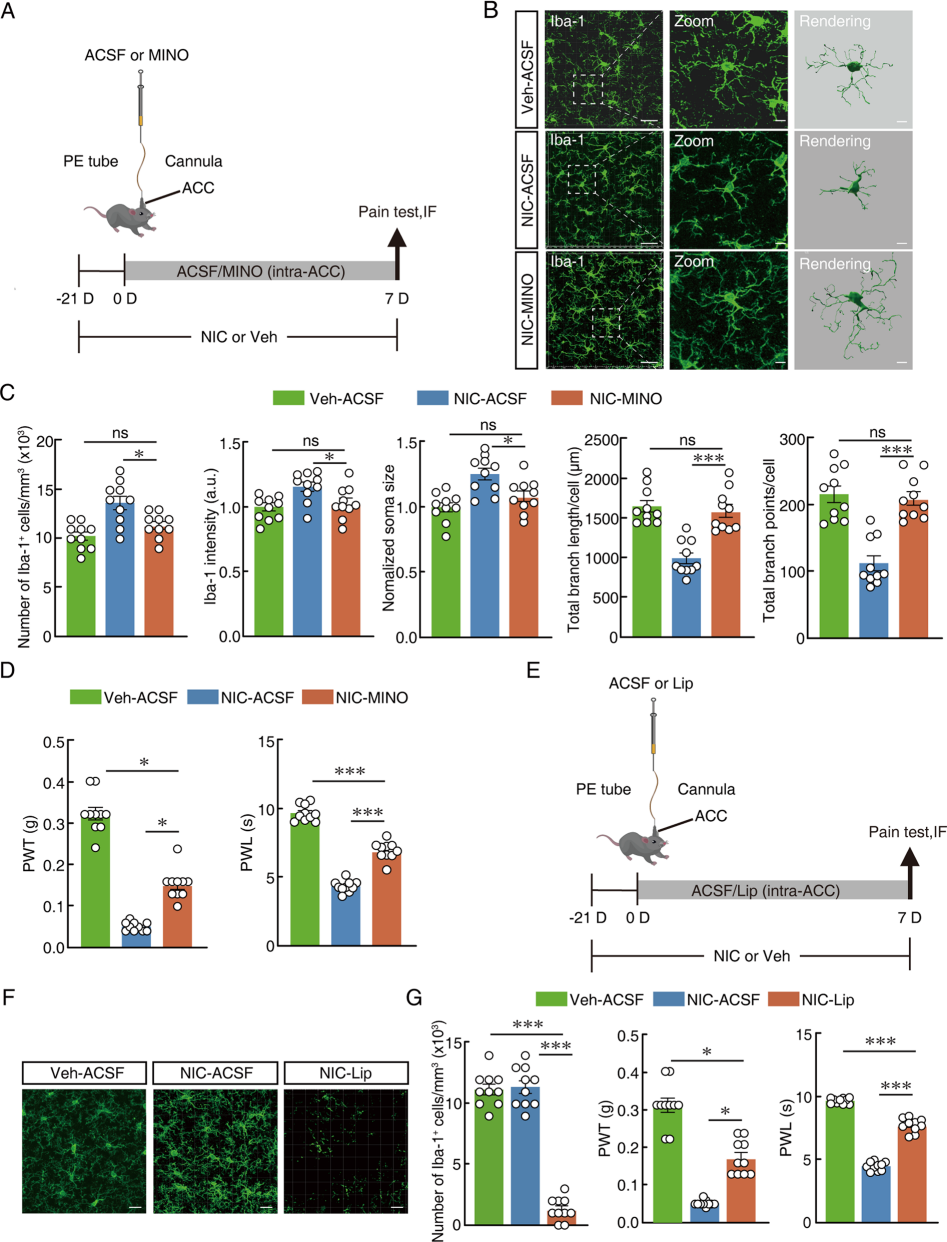
Reactive microglia in the ACC are required for nicotine-induced allodynia.** A** Schematic timeline of experiment. **B** Representative imaging of Iba-1-labelled microglia after intra-ACC ACSF in vehicle mice and intra-ACC ACSF or MINO in nicotine-treated mice. Scale bars, 40 μm (overview) and 10 μm (Zoom and Rendering). **C** Quantification of Iba-1^+^ number, intensity, soma size and Imaris-based semi-automatic quantification of Iba-1^+^ microglia morphometry in the ACC. **D** Changes of PWT and PWL in vehicle- and nicotine-treated mice with intra-ACC ACSF or MINO. **E** Schematic timeline of experiment. **F** Representative imaging of Iba-1-labelled microglia after intra-ACC ACSF in vehicle mice and intra-ACC ACSF or Lip in nicotine-treated mice. Scale bars, 40 μm (overview). **G** (Left) Quantification of Iba-1^+^ number in the ACC. (Middle and right) Changes of PWT and PWL in vehicle- and nicotine-treated mice with intra-ACC ACSF or Lip. Data were presented as mean ± SEM or Median (IQR). *p < 0.05; **p < 0.01; ***p < 0.001; *ns* not significant. Details of the statistical analyses are presented in Additional file 1: Table S1

To test the hypothesis that the hyperactivity of ACC microglia is necessary to sustain nicotine-induced allodynia, we aimed to investigate augmented pain-like response by suppressing and ablating reactive microglia by using intra-ACC administration of minocycline and liposome-clodronate, respectively (Fig. 2A). Minocycline has frequently been used to inhibit microglial activation [38]. Compared with ACSF control in the nicotine-treated mice, the morphological characteristics of microglia in nicotine-treated mice were significantly changed by intra-ACC minocycline (Fig. 2B). Our results showed that the number of Iba-1-labelled microglial cells was significantly reduced by intra-ACC minocycline, and minocycline significantly reversed the nicotine-induced reduction in both the number of microglial endpoints and process length (Fig. 2C). In addition, intra-ACC minocycline treatment resulted in a relief of nicotine-induced allodynia, which manifested by an obvious increase in mechanical and thermal thresholds (Fig. 2D). Intra-ACC minocycline increased the PWT and PWL in nicotine-treated mice compared with ACSF control. Next, liposome-clodronate was used for intra-ACC administration (Fig. 2E). Recent studies by Wang et al. [39] showed that liposome-clodronate produced a significant reduction in microglial cell numbers but had no effect on the number of neurons. After 7 days of liposome-clodronate intra-ACC administration, there was a strong microglial response in the ACC. Compared to ACSF control in nicotine-treated mice, the microglia in the ACC were almost completely ablated by intracranial administration of liposome-clodronate (Fig. 2F). In addition, compared to ACSF control, intracranial administration of liposome-clodronate relieved mechanical and thermal allodynia in nicotine-treated mice (Fig. 2G). These findings suggested that intracranial injection of minocycline and liposome-clodronate into ACC can reduce nicotine-induced allodynia.

### Nicotine-elicited reactive microglia do not exhibit inflammatory transcripts or phenotypes

**Fig. 3:**
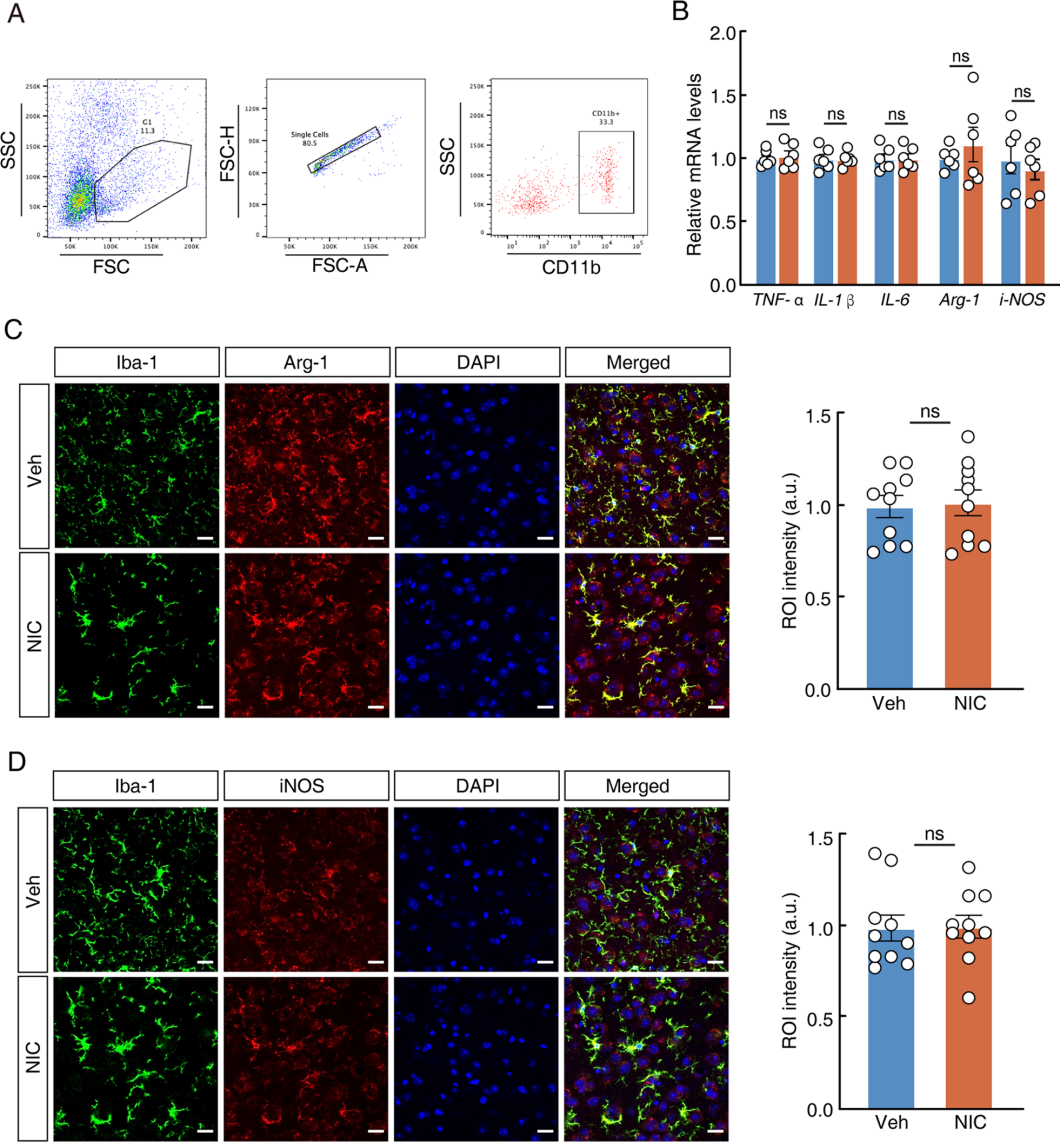
Nicotine-elicited reactive microglia do not exhibit an inflammatory transcripts or phenotypes. **A** Workflow diagram and the scheme of flow cytometry and cell sorting. SSC: side scatter; FSC: forward scatter; FSC-H: forward scatter-height; FSC-A: forward scatter-area. **B** qPCR analysis of *TNF-α*, *IL-1β*, *IL-6*, *Arg-1 and iNOS* mRNA from wild-type mice on day-28 post-administration of l-tartaric acid or nicotine. **C** Representative images (left) and intensity of ROI cells in the ACC (right). ROI (region of interest), Arg-1 and Iba-1 double positive cells. Scale bar, 20 μm. **D** Representative images (left) and intensity of ROI cells in the ACC (right). ROI (region of interest), iNOS and Iba-1 double positive cells. Scale bar, 20 μm. Data were presented as mean ± SEM or Median (IQR). *p < 0.05; **p < 0.01; ***p < 0.001; *ns* not significant. Details of the statistical analyses are presented in Additional file 1: Table S1

Microglia are essential for the early development of neuroinflammation and pain, and for promoting inflammation by releasing different proinflammatory cytokines including TNF-α, IL-1β and IL-6 [40]. To further test whether nicotine-induced allodynia causes classical inflammatory changes in microglia, we isolated ACC microglial cells by using fluorescence-activated cell sorting (Fig. 3A) and measured the changes in *TNF-α*, *IL-1β* and *IL-6* messenger RNA (mRNA) transcripts. The results showed no distinct difference in the levels of *TNF-α, IL-1β* and *IL-6* mRNA in nicotine-treated mice compared with vehicle control (Fig. 3B). To further validate this, other inflammation-related markers, including iNOS and Arg-1, were examined by using immunofluorescence staining of brain slices. Our results showed that neither Arg-1 nor iNOS showed significant changes in Iba1-labelled microglia between the nicotine and vehicle groups (Fig. 3C, D). Due to the nonmicroglial expression found in the brain slices, we focused on cellular *Arg-1* and *iNOS* mRNA levels in isolated microglia. There was no significant difference in microglial *Arg-1* or *iNOS* between the groups (Fig. 3B). In brief, these findings showed that nicotine did not cause inflammation with reactive microglia in the ACC in mice.

### Hyperactivity of ACC pyramidal neurons simultaneously develops in chronic nicotine-treated mice

**Fig. 4:**
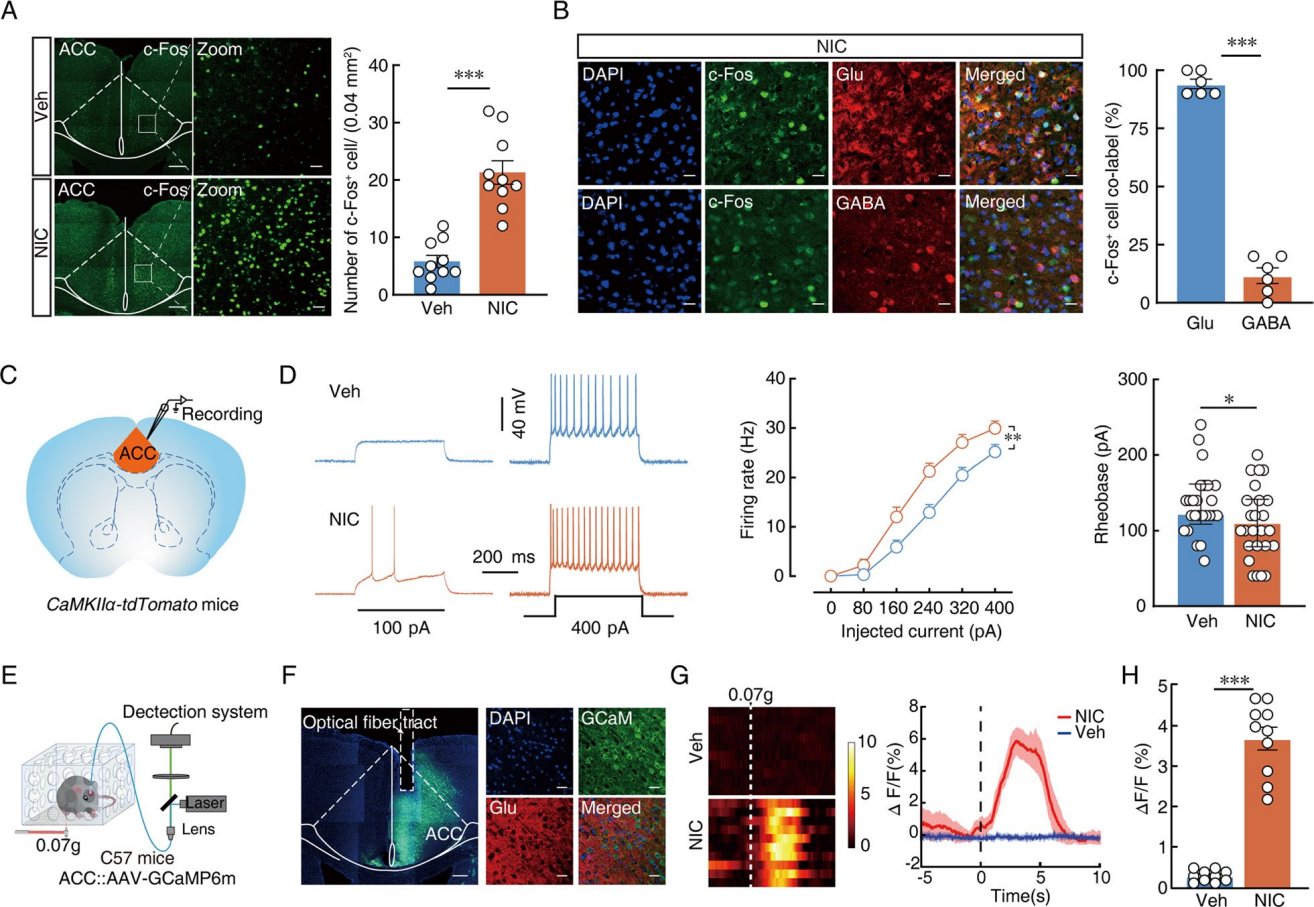
Hyperactivity of ACC pyramidal neurons simultaneously develops in chronic nicotine-treated mice. **A** (Left) Bilateral distribution of c-Fos^+^ neurons in the ACC in vehicle- and nicotine-treated mice after 28 days. Scale bar, 200 μm (left), 20 μm (right); (Right) number of c-Fos^+^ cells per 0.04mm^2^ area in the ACC. **B** showing that c-Fos co-labeled neurons within the ACC on day 28 after nicotine-treated were mainly co-localized with glutamatergic immunofluorescence and statistics data. Scale bars, 20 μm. **C** Schematic for viral injection and whole-cell recordings. **D** Sample traces (left) and data (middle and right) of action potentials and summarize of rheobase of vehicle- and nicotine-treated mice. **E** Schematic and timelines of fiber photometry. **F** Representative imaging of GCaMP6m viral expression in the ACC glutamatergic neurons 3 weeks after viral injection. The boxed region (dashed lines) represents the site of implantable fiber optic cannula. Scale bars, 200 μm (left), 20 μm (right). **G** The heatmaps (left) and mean (right) show that Ca^2+^ signals rapidly increased in nicotine-treated mice compared with vehicle mice after 28 days. The colored bar on the right indicates ΔF/F (%). **H** Average ΔF/F of ACC^GCaMP6m^ signals in vehicle- and nicotine-treated mice. Data were presented as mean ± SEM or Median (IQR). *p < 0.05; **p < 0.01; ***p < 0.001; *ns* not significant. Details of the statistical analyses are presented in Additional file 1: Table S1

Microglia may be involved in the mechanism of pain sensitization by promoting hyperexcitation of innervated sensory neurons [13, 19]. We thus observed whether the alteration of ACC pyramidal neurons is tightly related to nicotine-induced allodynia. To investigate whether chronic nicotine can activate ACC pyramidal neurons, immunofluorescent staining was performed on c-Fos, a marker of neuronal activity in the ACC. The number and intensity of c-Fos in nicotine-treated mice increased significantly compared to vehicle treatment (Fig. 4A). Subsequent immunofluorescence results showed that more than 90% of c-Fos colocalized with glutamatergic neurons (Fig. 4B). To confirm these results, we used ACC brain slices from CamKIIα-Cre:: Ai14 mice, in which tdTomato specifically labelled glutamatergic neurons (Fig. 4C). Whole-cell patch clamp results showed that, compared with vehicle control mice, nicotine-treated mice displayed increased excitability of ACC glutamatergic neurons, presenting an increase in spike number and a decrease in rheobase (Fig. 4D). Additionally, to explore whether ACC glutamatergic neurons are sensitive to subthreshold stimuli, rAAV-CaMKIIα-GCaMP6m-EGFP, a virus that expressed calcium indicators in glutamatergic neurons after 3 weeks, was injected into the ACC for fibre photometer recordings of nicotine-treated mice (Fig. 4E, F). Compared with the vehicle control, calcium signals increased rapidly after stimulation with a 0.07 g von Frey filament on the paws of nicotine-treated mice (Fig. 4G). Collectively, these data indicate that glutamatergic neurons are activated in chronic nicotine-treated mice.

### Inhibition of ACC pyramidal neurons reduces nicotine-induced allodynia and microglia activation

**Fig. 5:**
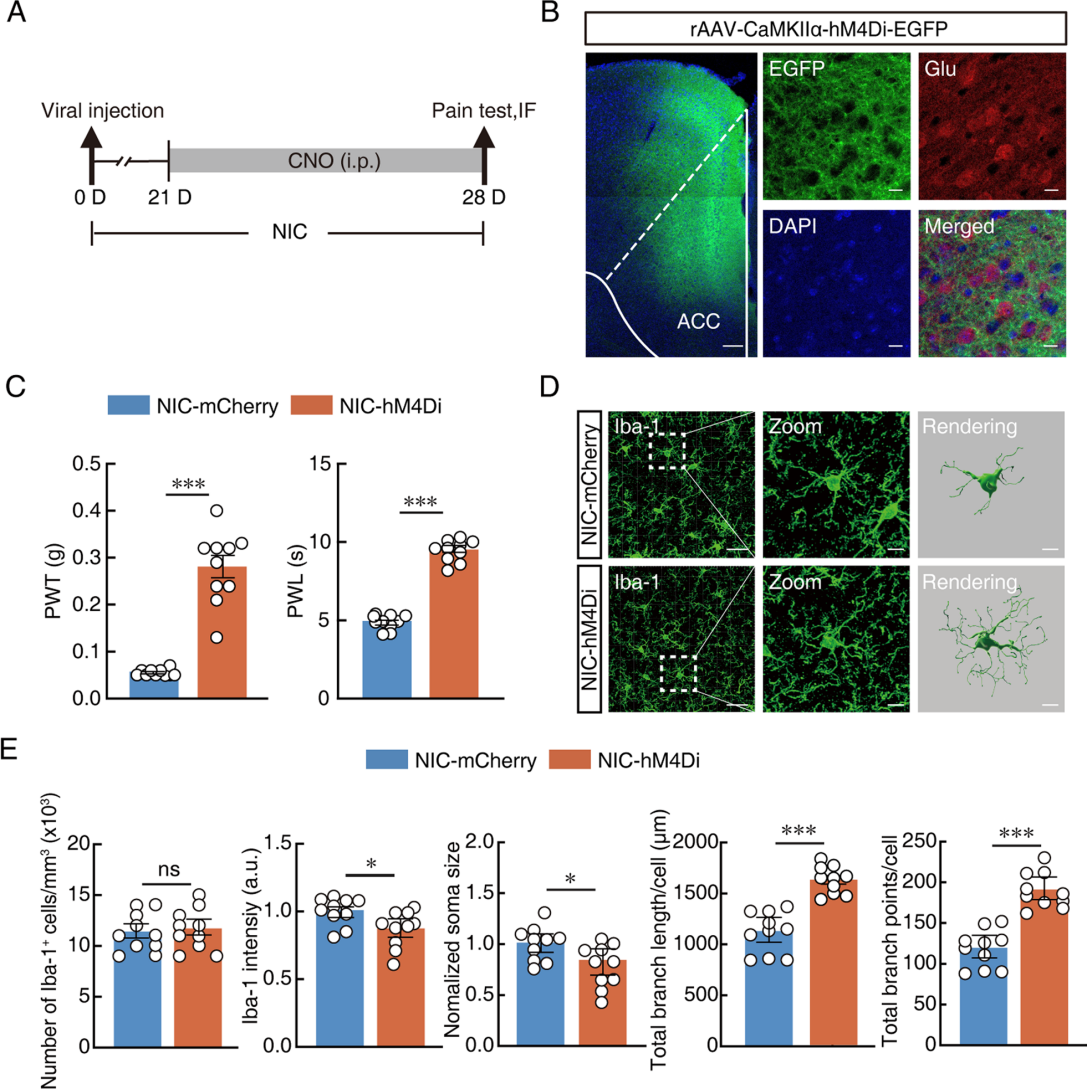
Inhibition of ACC pyramidal neurons reduces nicotine-induced allodynia and microglia activation. **A** Schematic timeline of experiment. **B** Representative imaging of hM4Di viral expression within glutamatergic neurons in the ACC 3 weeks after viral injection. Scale bars, 200 µm (left) and 20 µm (right). **C** Changes of PWT and PWL in nicotine-treated mice after inhibition of glutamatergic neurons. **D** Representative imaging and 3D remodeling of Iba-1-labeled microglia in the ACC in response to mCherry or hM4Di in nicotine-treated mice. **E** Quantification of Iba-1^+^ number, intensity, soma size and Imaris-based semi-automatic quantification of Iba-1^+^ microglia morphometry in the ACC. Scale bars, 40 μm (overview) and 10 μm (Zoom and Rendering). Data were presented as mean ± SEM or Median (IQR). *p < 0.05; **p < 0.01; ***p < 0.001; *ns* not significant. Details of the statistical analyses are presented in Additional file 1: Table S1

To further understand the reciprocal interactions between neurons and microglia, chemogenetic approach was used to inhibit glutamatergic neurons, and the impact on the microglial response and allodynia over time was observed (Fig. 5A). rAAV-CaMKIIα-hM4Di-mCherry was injected into nicotine-treated mice and successfully expressed in ACC glutamatergic neurons after 3 weeks, and after selective inhibition of ACC glutamatergic neurons by intraperitoneal injection of CNO, both mechanical and thermal pain thresholds increased (Fig. 5B, C). Finally, to investigate whether there is an association between glutamatergic neuronal overactivity and microglial activation, we observed changes in microglial cells after consecutive intraperitoneal administration of CNO. Morphological analysis of microglial cells showed that activation of microglia was effectively eliminated due to chemical inhibition of glutamatergic neurons (Fig. 5D, E). In brief, these findings showed that the activation of ACC glutamatergic neurons may precede microglial activation in nicotine-treated mice. By inhibiting glutamatergic neurons, the activation of microglia was effectively eliminated and nicotine-induced pain thresholds were also increased.

### Fractalkine signalling mediates ACC neuron-microglia interactions in nicotine-induced allodynia

**Fig. 6:**
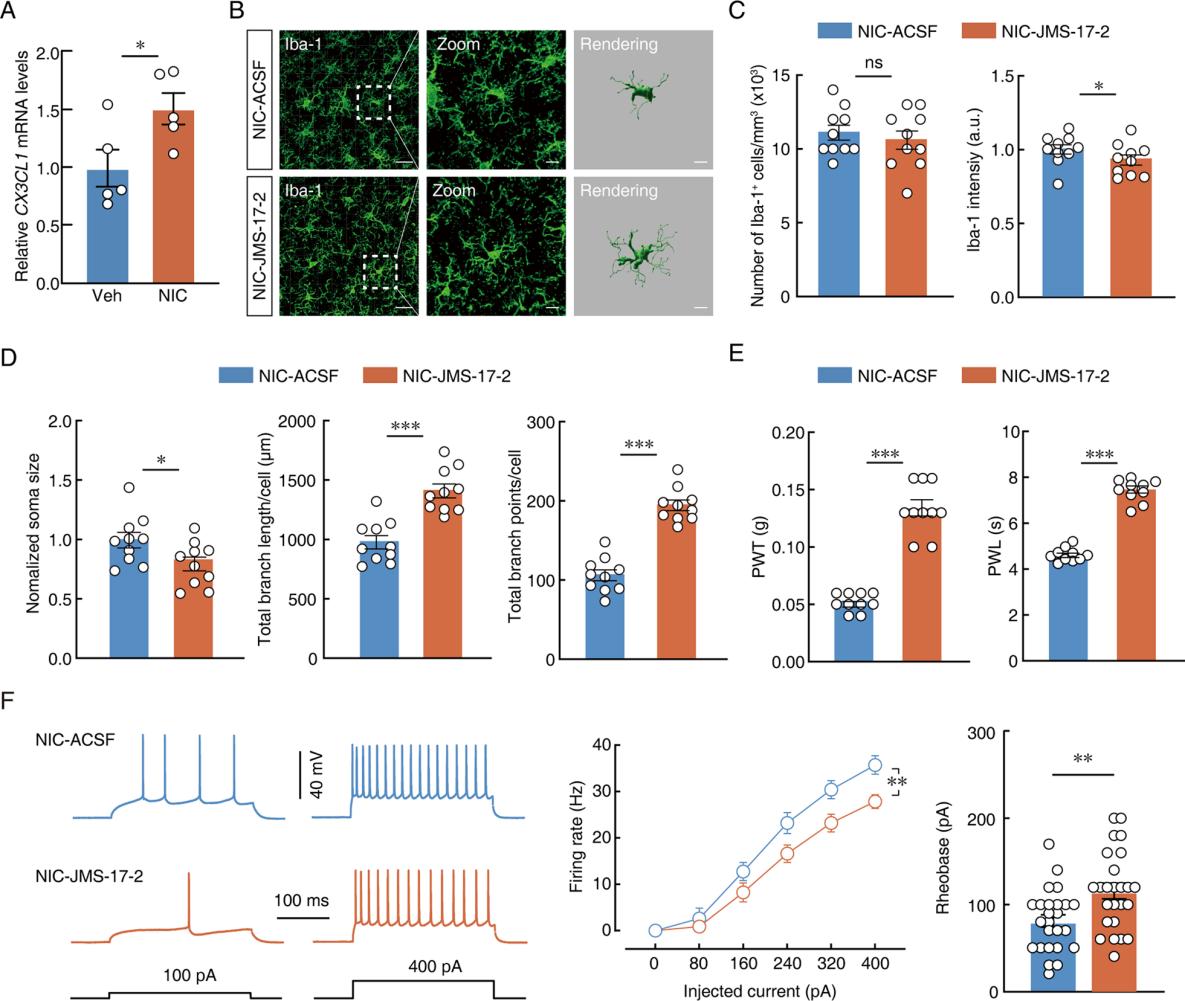
Fractalkine signalling mediates ACC neuron-microglia interactions in nicotine-induced allodynia.** A** qPCR analysis of *CX3CL1* mRNA in vehicle- or nicotine-treated mice after 28 days. **B** Representative imaging of Iba-1-labeled microglia after intra-ACC ACSF or JMS-17-2 in nicotine-treated mice. Scale bars, 40 μm (overview) and 10 μm (Zoom and Rendering). **C**, **D** Quantification of Iba-1^+^ number, intensity, soma size and Imaris-based semi-automatic quantification of Iba-1^+^ microglia morphometry in the ACC. **E** Changes of PWT and PWL in nicotine-treated mice with intra-ACC ACSF or JMS-17-2.** F** Sample traces (left) and data of action potentials (middle) and summarize of rheobase (right) of intra-ACC ACSF or JMS-17-2. Data were presented as mean ± SEM or Median (IQR). *p < 0.05; **p < 0.01; ***p < 0.001; *ns* not significant. Details of the statistical analyses are presented in Additional file 1: Table S1

Chemokines play a key role in mediating neuron-microglia communication, leading to increased pain perception [41]. Fractalkine is unique in the structure of the chemokine family, and its receptors are expressed both in the CNS and peripheral nerves, as well as in endothelial cells and lymphocytes. CX3CL1 (fractalkine)-CX3CR1 signalling represents the most important communication channel between neurons and microglia. To test the hypothesis that this pathway is involved in nicotine-induced allodynia changes, qPCR was used to detect the change in CX3CL1 content in the ACC. CX3CL1 expression was increased in nicotine-treated mice compared with vehicle-treated mice (Fig. 6A). CX3CL1 signalling relied on binding with downstream CX3CR1. JMS-17-2 acted as a selective small molecule inhibitor of CX3CR1 by intra-ACC injection. Our results showed that after intracranial injection of JMS-17-2 in nicotine-treated mice, there was a significant change in microglial morphology in the ACC, manifested by a decrease in the intensity of Iba-1-labelled microglia, accompanied by a decrease in the number of endpoints and the length of the process (Fig. 6B–D). Compared to intra-ACSF control, intra-ACC injection of JMS-17-2 alleviated nicotine-induced chronic allodynia (Fig. 6E). To verify whether inhibition of CX3CR1 can affect the excitability of glutamatergic neurons, ACC brain slices were prepared and incubated with the antagonist JMS-17-2 (10 nM) for 30 min at 37 °C, and then a patch-clamp electrophysiological experiment was performed. Electrophysiological results showed that, compared with nicotine-treated mice, the current-elicited action potentials decreased and rheobase increased in JMS-17-2-treated mice (Fig. 6F). These results suggest that microglia may maintain neuronal excitability through the CX3CL1-CX3CR1 signalling pathway.

## Discussion

Nicotine, the main addictive ingredient in cigarettes, is thought to produce a temporary analgesic property when transiently administered [42, 43]. However, epidemiologic evidence shows that long-term smoking is a risk factor for chronic pain. A higher incidence and severity of pain experience has been found in regular smokers [44–46]. Our study suggests that chronic nicotine exposure causes nociceptive hypersensitivity in mice, and the neuroadaptation in the ACC, which manifests as hypersensitivity of glutamate neurons and activation of microglial cells, develops in response to chronic nicotine exposure. In addition, reciprocal communication between microglia and neurons occurred and further, at least in part, contributed to the allodynia induced by chronic nicotine.

The family of nAChRs shows wide distribution in the central and peripheral nervous systems and is involved in numerous processes, including arousal, sleep, anxiety, cognition, and pain [47]. Nicotine addiction is a complex process. In addition to the release of VTA dopamine indirectly via stimulation of nAChRs, nicotine dependence may be related to neuronal interactions, transmitter release, receptor sensitization and desensitization [48, 49]. The ACC is known to be involved in pain and is associated with negative emotional regulation. Neuroimaging studies also support a functional role of the ACC in addiction-related neural networks [25]. A high density of neuronal nAChR expression in the human ACC suggests that nicotine may activate ACC neurons by direct action [50].

Microglial cells are highly specialized resident immune cells in the brain that detect and respond to interference by changing their morphology in response to the type of injury [51, 52]. Recent studies have shown that microglia in NAc are key mediators of nicotine dependence [10], but little is known regarding the role of ACC microglia in nicotine-elicited allodynia and their phenotypic changes in response to chronic nicotine exposure. Our study showed that chronic nicotine-induced allodynia is associated with concomitant reactive microglia in the ACC. In addition to antimicrobial activity, minocycline is well known for its anti-inflammatory and neuroprotective effects. It has been used frequently to suppress microglial activation in disease context. However, it has also been reported that minocycline has potential neuroprotective properties. In our previous study, by using a combination of ex vivo brain slice electrophysiology and PLX3397-induced microglia depletion, we concluded that a concentration of 50 µM minocycline ex vivo exerts an inhibitory effect on glutamatergic neurons in a microglia-dependent manner, and the dosage of 10 mg/ml used for intra-ACC administration of minocycline in vivo was calculated accordingly [19]. Similarly, we employed the dosage of 10 mg/ml minocycline in the present study.

Indeed, similar findings have also been reported in prior literature, showing chronic nicotine does not elicit a classical inflammatory response despite microglial morphological changes in the NAc [9]. These findings in animal studies are consistent with many prior human studies demonstrating that smokers did not experience typical inflammatory responses compared with nonsmokers [53]. This finding is conflicting with the established view of a causal relationship between reactive microglia and inflammation. Thus it’s interesting to know whether and how reactive microglia contribute to ACC neuronal hyperactivity in nicotine-induced allodynia.

Furthermore, an interaction between ACC neurons and microglia is involved in nicotine-induced allodynia, and this reciprocal process may be mediated through the CX3CL1 signalling cascade [54]. We suspect that ACC glutamatergic neurons are highly excited under chronic nicotine and then induce activation of microglia by the increased expression of fractalkine (CX3CL1), which acts on microglial receptor CX3CR1, resulting in altered signalling pathways that promote and maintain nociceptive hypersensitivity.

In addition to their function in immunity and inflammation, microglial cells are involved in synaptic function plasticity, and microglial CX3CR1 signalling has been demonstrated to mediate developmental synaptic pruning through the neuronal ligand CX3CL1. Therefore, microglial activation under chronic nicotine within the ACC may enhance the development of aberrant synaptic connections and plasticity underlying nicotine dependency. However, one limitation of our study is that we did not work further on microglial synaptic pruning.

Our experimental evidence showed that nicotine-induced allodynia in mice may be due to, at least in part, hyperexcitability of ACC glutamatergic neurons and activation of microglia. Therefore, one of the central mechanisms by which microglia sustain ACC neuronal hyperactivity underlies nicotine-induced allodynia in mice.

## Supplementary Information

**Additional file 1**: **Table S1** Detailed statistical information.

## Data Availability

The datasets used and/or analysed during the current study are available from the corresponding author on reasonable request.

## Abbreviations

ACC: Anterior cingulate cortex
nAChRs: Nicotinic acetylcholine receptors
CNS: Central nervous system
NAc: Nucleus accumbens
MRI: Magnetic resonance imaging
PWT: Paw withdrawl threthold
PWL: Latency to withdrawl
ACSF: Artificial cerebrospinal fluid
MINO: Minocycline
Lip: Liposome-clodronate
CNO: Clozapine *N*-oxide
qPCR: Quantitative real-time PCR
IF: Immunofluorescence
