# Supplementary material for: Microglia sustain anterior cingulate cortex neuronal hyperactivity in nicotine-induced pain
Source: J Neuroinflammation. 2023 Mar 21;20:81. doi: 10.1186/s12974-023-02767-0 (PMC10031886; doi:10.1186/s12974-023-02767-0)
Supplement: Supplementary file 1 — Additional file 1: Table S1 Detailed statistical information. [file 12974_2023_2767_MOESM1_ESM.pdf]

Table S1 Detailed statistical information

Figure	Normality test	n/group	conditions	Statistical Method	comparison	Mean ± SEM/Median (IQR)	Main P-Value	F or t Value
1.B	Yes	Veh, n=10 mice; NIC, n=10 mice	PWT	Two-tailed unpaired *t*-test	Veh vs NIC	0.365 ± 0.020<br>0.047 ± 0.003	<0.001	t_{18}=15.49
	Yes	Veh, n=10 mice; NIC, n=10 mice	PWL	Two-tailed unpaired *t*-test	Veh vs NIC	10.38 ± 0.161<br>4.458 ± 0.119	<0.001	t_{18}=29.61
1.E	Yes	Veh, n=10 slices from 5mice; NIC, n=10 slices from 5 mice	Number	Two-tailed unpaired *t*-test	Veh vs NIC	13.10 ± 0.640<br>13.40 ± 0.702	0.7559	t_{18}=0.3156
	Yes	Veh, n=10 slices from 5 mice; NIC, n=10 slices from 5 mice	Intensity	Two-tailed unpaired *t*-test	Veh vs NIC	1.00 ± 0.042<br>1.12 ± 0.031	0.0351	t_{18}=2.279
1.F	Yes	Veh, n=10 slices from 5 mice; NIC, n=10 slices from 5 mice	Size	Two-tailed unpaired *t*-test	Veh vs NIC	1.00 ± 0.061<br>1.17 ± 0.047	0.0137	t_{18}=2.733

	Yes	Veh, n=10 slices from 5 mice; NIC, n=10 slices from 5 mice	Length	Two-tailed unpaired *t*-test	Veh vs NIC	1684 ± 78.81<br>1118 ± 56.16	<0.001	t_{18}=5.854
	Yes	Veh, n=10 slices from 5 mice; NIC, n=10 slices from 5 mice	Points	Two-tailed unpaired *t*-test	Veh vs NIC	189.4 ± 5.55<br>121.7 ± 6.05	<0.001	t_{18}=8.249
1.H	Yes	Veh, n=10 slices from 5 mice; NIC, n=10 slices from 5 mice	Number	Two-tailed unpaired *t*-test	Veh vs NIC	14.40 ± 0.50<br>14.10 ± 0.50	0.6774	t_{18}=0.4229
	Yes	Veh, n=10 slices from 5 mice; NIC, n=10 slices from 5 mice	Intensity	Two-tailed unpaired *t*-test	Veh vs NIC	1.00 ± 0.04<br>1.27 ± 0.05	0.0011	t_{18}=3.864
	No	Veh, n=10 slices from 5 mice; NIC, n=10 slices from 5 mice	Size	Mann-Whitney U test	Veh vs NIC	1.00 (0.87, 1.12)<br>1.17 (1.11, 1.22)	0.0451	U=23.50
	Yes	Veh, n=10 slices from 5 mice; NIC, n=10 slices from 5 mice	Length	Two-tailed unpaired *t*-test	Veh vs NIC	1658 ± 69.53<br>1129 ± 52.37	<0.001	t_{18}=6.075
	Yes	Veh, n=10 slices	Points	Two-tailed	Veh vs NIC	196.4 ± 6.50	<0.001	t_{18}=8.718

		from 5 mice; NIC, n=10 slices from 5 mice		unpaired *t*-test		120.9 ± 5.72		
2.C	Yes	Veh-ACSF, n=10 slices from 5 mice; NIC-ACSF, n=10 slices from 5 mice; NIC-MINO, n=10 slices from 5 mice	Number	One-way RM ANOVA	Veh-ACSF vs NIC-MINO	10.30 ± 0.42	0.3162	$F_{(2, 27)}$=10.89
						13.70 ± 0.70		
					NIC-ACSF vs NIC-MINO	11.40 ± 0.40	0.0123	
	Yes	Veh-ACSF, n=10 slices from 5 mice; NIC-ACSF, n=10 slices from 5 mice; NIC-MINO, n=10 slices from 5 mice	Intensity	One-way RM ANOVA	Veh-ACSF vs NIC-MINO	1.00 ± 0.03	>0.9999	$F_{(2, 27)}$=4.597
						1.08 ± 0.04		
					NIC-ACSF vs NIC-MINO	0.95 ± 0.04	0.0366	
	Yes	Veh-ACSF, n=10 slices from 5 mice; NIC-ACSF, n=10 slices from 5 mice; NIC-MINO, n=10	Size	One-way RM ANOVA	Veh-ACSF vs NIC-MINO	1.00 ± 0.04	0.3142	$F_{(2, 27)}$=10.36
						1.23 ± 0.05		
					NIC-ACSF vs NIC-MINO	1.06 ± 0.04	0.0159	

		slices from 5 mice						
	Yes	Veh-ACSF, n=10 slices from 5 mice; NIC-ACSF, n=10 slices from 5 mice; NIC-MINO, n=10 slices from 5 mice	Length	One-way RM ANOVA	Veh-ACSF vs NIC-MINO	1660 ± 73.47	0.8476	$F_{(2, 27)}$=23.81
						1000 ± 68.42		
					NIC-ACSF vs NIC-MINO	1601 ± 81.94	<0.001	
	Yes	Veh-ACSF, n=10 slices from 5 mice; NIC-ACSF, n=10 slices from 5 mice; NIC-MINO, n=10 slices from 5 mice	Points	One-way RM ANOVA	Veh-ACSF vs NIC-MINO	217.1 ± 12.45	0.9211	$F_{(2, 27)}$=26.48
						113.1 ± 11.26		
					NIC-ACSF vs NIC-MINO	210.9 ± 10.18	<0.001	
2.D	No	Veh-ACSF, n=10 mice; NIC-ACSF, n=10 mice; NIC-MINO, n=10 mice	PWT	Kruskal-Wallis test	Veh-ACSF vs NIC-MINO	0.32 (0.290, 0.340)	0.0340	H=26.04
						0.050 (0.040, 0.060)		
					NIC-ACSF vs NIC-MINO	0.160 (0.130, 0.160)	0.0305	
	Yes	Veh-ACSF, n=10 mice;	PWL	One-way RM ANOVA	Veh-ACSF vs NIC-MINO	9.74 ± 0.19	<0.001	$F_{(2, 27)}$=222.1

		NIC-ACSF, n=10 mice; NIC-MINO, n=10 mice				4.37 ± 0.14		
					NIC-ACSF vs NIC-MINO	6.98 ± 0.21	<0.001	
2.G	Yes	Veh-ACSF, n=10 slices from 5 mice; NIC-ACSF, n=10 slices from 5 mice; NIC-Lip, n=10 slices from 5 mice	Number	One-way RM ANOVA	Veh-ACSF vs NIC-Lip	11.20 ± 0.44	<0.001	$F_{(2, 27)}$=179.7
						11.40 ± 0.52		
					NIC-ACSF vs NIC-Lip	1.30 ± 0.30	<0.001	
	No	Veh-ACSF, n=10 mice; NIC-ACSF, n=10 mice; NIC-Lip, n=10 mice	PWT	Kruskal-Wallis test	Veh-ACSF vs NIC-Lip	0.320 (0.300, 0.340)	0.0405	H=25.96
						0.050 (0.040, 0.053)		
					NIC-ACSF vs NIC-Lip	0.160 (0.130, 0.218)	0.0260	
	Yes	Veh-ACSF, n=10 mice; NIC-ACSF, n=10 mice; NIC-Lip, n=10 mice	PWL	One-way RM ANOVA	Veh-ACSF vs NIC-Lio	9.73 ± 0.07	<0.001	$F_{(2, 27)}$=441.5
						4.51 ± 0.11		
					NIC-ACSF vs NIC-Lip	7.78 ± 0.17	<0.001	
3.B	Yes	Veh, n=6 mice;	TNF-α	Two-tailed	Veh vs NIC	1.00 ± 0.03	0.6852	t_{10}=0.4174

		NIC, n=6 mice		unpaired *t*-test		1.02 ± 0.04		
	Yes	Veh, n=6 mice; NIC, n=6 mice	IL-1β	Two-tailed unpaired *t*-test	Veh vs NIC	1.00 ± 0.04<br>1.00 ± 0.02	0.9385	t_{10}=0.0791
	Yes	Veh, n=6 mice; NIC, n=6 mice	IL-6	Two-tailed unpaired *t*-test	Veh vs NIC	1.00 ± 0.04<br>1.00 ± 0.04	0.9521	t_{10}=0.0616
	Yes	Veh, n=6 mice; NIC, n=6 mice	Arg-1	Two-tailed unpaired *t*-test	Veh vs NIC	1.00 ± 0.04<br>1.12 ± 0.14	0.4580	t_{10}=0.7720
	Yes	Veh, n=6 mice; NIC, n=6 mice	iNOS	Two-tailed unpaired *t*-test	Veh vs NIC	1.00 ± 0.11<br>0.91 ± 0.08	0.5828	t_{10}=0.5677
3.C	Yes	Veh, n=6 mice; NIC, n=6 mice	ROI	Two-tailed unpaired *t*-test	Veh vs NIC	1.00 ± 0.06<br>1.02 ± 0.07	0.8322	t_{18}=0.2150
3.D	Yes	Veh, n=6 mice; NIC, n=6 mice	ROI	Two-tailed unpaired *t*-test	Veh vs NIC	1.00 ± 0.07<br>1.01 ± 0.06	0.9403	t_{18}=0.0760
4.A	Yes	Veh, n=10 slices from 3 mice; NIC, n=10 slices from 3 mice	Number of c-Fos+ cell	Two-tailed unpaired *t*-test	Veh vs NIC	5.80 ± 1.09<br>21.30 ± 2.06	<0.001	t_{18}=6.659
4.B	No	Veh, n=6 slices from 3 mice; NIC, n=6 slices from 3 mice	Co-label	Mann-Whitney U test	Glu vs GABA	92.50 (90, 100)<br>12.5 (3.75, 20)	0.0022	U=0
4.D	Yes	Veh, n=25 cells from 3 mice; NIC, n=25 cells from 3 mice	Firing rate	Two-way RM ANOVA	Veh vs NIC	10.79 ± 4.30<br>15.40 ± 5.18	0.0047	$F_{(5,144)}$=3.543

	Yes	Veh, n=25 cells from 3 mice; NIC, n=25 cells from 3 mice	Rheobase	Two-tailed unpaired *t*-test	Veh vs NIC	135.2 ± 8.27<br>108.8 ± 9.53	0.0417	t_{48}=2.093
4.H	Yes	Veh, n=4 mice; NIC, n=3 mice	ΔF/F (%)	Two-tailed unpaired *t*-test	Veh vs NIC	0.30 ± 0.05<br>3.71 ± 0.28	<0.001	t_{18}=11.9
5.C	Yes	NIC-mCherry, n=10 mice; NIC-hM4Di, n=10 mice	PWT	Two-tailed unpaired *t*-test	NIC-mCherry vs NIC-hM4Di	0.055 ± 0.002<br>0.279 ± 0.024	<0.001	t_{18}=9.324
	Yes	NIC-mCherry, n=10 mice; NIC-hM4Di, n=10 mice	PWL	Two-tailed unpaired *t*-test	NIC-mCherry vs NIC-hM4Di	4.89 ± 0.15<br>9.45 ± 0.22	<0.001	t_{18}=17.29
5.E	Yes	NIC-mCherry, n=10 slices from 5 mice; NIC-hM4Di, n=10 slices from 5 mice	Number	Two-tailed unpaired *t*-test	NIC-mCherry vs NIC-hM4Di	11.30 ± 0.60<br>11.60 ± 0.64	0.7349	t_{18}=0.3439
	Yes	NIC-mCherry, n=10 slices from 5 mice; NIC-hM4Di, n=10 slices from 5 mice	Intensity	Two-tailed unpaired *t*-test	NIC-mCherry vs NIC-hM4Di	1.00 ± 0.03<br>0.87 ± 0.04	0.0200	t_{18}=2.551

	Yes	NIC-mCherry, n=10 slices from 5 mice; NIC-hM4Di, n=10 slices from 5 mice	Size	Two-tailed unpaired *t*-test	NIC-mCherry vs NIC-hM4Di	1.00 ± 0.05<br>0.79 ± 0.07	0.0239	t_{18}=2.468
	Yes	NIC-mCherry, n=10 slices from 5 mice; NIC-hM4Di, n=10 slices from 5 mice	Length	Two-tailed unpaired *t*-test	NIC-mCherry vs NIC-hM4Di	1119 ± 65.15<br>1627 ± 42.41	<0.001	t_{18}=6.531
	Yes	NIC-mCherry, n=10 slices from 5 mice; NIC-hM4Di, n=10 slices from 5 mice	Points	Two-tailed unpaired *t*-test	NIC-mCherry vs NIC-hM4Di	118.2 ± 7.34<br>189.6 ± 6.88	<0.001	t_{18}=7.099
6.A	Yes	Veh, n=5 mice; NIC, n=5 mice	CX3CL1	Two-tailed unpaired *t*-test	Veh vs NIC	1.000 ± 0.16<br>1.516 ± 0.14	0.0404	t_8=2.443
6.C	Yes	NIC-ACSF, n=10 slcies from 5 mice; NIC-JMS-17-2, n=10 slices from 5 mice	Number	Two-tailed unpaired *t*-test	NIC-ACSF vs NIC-JMS-17-2	11.10 ± 0.50<br>10.60 ± 0.62	0.5388	t_{18}=0.6266

	Yes	NIC-ACSF, n=10 slices from 5 mice; NIC-JMS-17-2, n=10 slices from 5 mice	Intensity	Two-tailed unpaired *t*-test	NIC-ACSF vs NIC-JMS-17-2	1.00 ± 0.03<br>0.90 ± 0.03	0.0419	t_{18}=2.190
6.D	Yes	NIC-ACSF, n=10 slices from 5 mice; NIC-JMS-17-2, n=10 slices from 5 mice	Size	Two-tailed unpaired *t*-test	NIC-ACSF vs NIC-JMS-17-2	1.00 ± 0.07<br>0.81 ± 0.06	0.0347	t_{18}=2.284
	Yes	NIC-ACSF, n=10 slices from 5 mice; NIC-JMS-17-2, n=10 slices from 5 mice	Length	Two-tailed unpaired *t*-test	NIC-ACSF vs NIC-JMS-17-2	977.2 ± 55.59<br>1410 ± 58.05	<0.001	t_{18}=5.380
	Yes	NIC-ACSF, n=10 slices from 5 mice; NIC-JMS-17-2, n=10 slices from 5 mice	Points	Two-tailed unpaired *t*-test	NIC-ACSF vs NIC-JMS-17-2	106.0 ± 6.96<br>194.3 ± 6.73	<0.001	t_{18}=9.118
6.E	Yes	NIC-ACSF, n=10 mice;	PWT	Two-tailed unpaired *t*-test	NIC-ACSF vs NIC-JMS-17-2	0.05 ± 0.002	<0.001	t_{18}=10.90

		NIC-JMS-17-2, n=10 mice				0.13 ± 0.007		
	Yes	NIC-ACSF, n=10 mice; NIC-JMS-17-2, n=10 mice	PWL	Two-tailed unpaired *t*-test	NIC-ACSF vs NIC-JMS-17-2	4.63 ± 0.09	<0.001	t_{18}=15.62
						7.43 ± 0.15		
6.F	Yes	NIC-ACSF, n=25 cells from 3 mice; NIC-JMS-17-2, n=25 cells from 3 mice	Firing rate	Two-way RM ANOVA	NIC-ACSF vs NIC-JMS-17-2	17.45 ± 6.00	0.0086	$F_{(5,144)}$=3.228
						12.79 ± 4.75		
	Yes	NIC-ACSF, n=25 cells from 3 mice; NIC-JMS-17-2, n=25 cells from 3 mice	Rheobase	Two-tailed unpaired *t*-test	NIC-ACSF vs NIC-JMS-17-2	81.20 ± 7.17	0.0040	t_{48}=3.020
						116 ± 9.02		
